# Structural correlates of active-staining following magnetic resonance microscopy in the mouse brain

**DOI:** 10.1016/j.neuroimage.2011.01.082

**Published:** 2011-06-01

**Authors:** Jon O. Cleary, Frances K. Wiseman, Francesca C. Norris, Anthony N. Price, ManKin Choy, Victor L.J. Tybulewicz, Roger J. Ordidge, Sebastian Brandner, Elizabeth M.C. Fisher, Mark F. Lythgoe

**Affiliations:** a Centre for Advanced Biomedical Imaging, Department of Medicine and Institute of Child Health, University College London, 72 Huntley Street, London, WC1E 6DD, UK; b Department of Medical Physics and Bioengineering, University College London, Gower Street, London, WC1E 6BT, UK; c Department of Neurodegenerative Disease, Institute of Neurology, University College London, Queen Square, London, WC1N 3BG, UK; d Centre for Mathematics and Physics in the Life Sciences and Experimental Biology (CoMPLEX), University College London, Gower Street, London, WC1E 6BT, UK; e MRC National Institute for Medical Research, The Ridgeway, Mill Hill, London, NW7 1AA, UK; f Wellcome Trust Advanced MRI Group, University College London, 8–11 Queen Square, Queen Square, London, WC1N 3BG, UK; g Division of Neuropathology and Department of Neurodegenerative Disease, Institute of Neurology, University College London, Queen Square, London, WC1N 3BG, UK

**Keywords:** MRI, magnetic resonance imaging, CNS, central nervous system, Gd-DTPA, gadolinium-diethylene-triamine-pentaacetic acid, SNR, signal-to-noise ratio, CNR, contrast-to-noise ratio, FOV, field of view, NSA, number of signal averages, TE, echo time, TR, repetition time, FA, flip angle, Magnetic resonance microscopy, Mouse brain phenotyping, Active staining, Mouse brain histology, Immunohistochemistry, Myelin, Grey matter

## Abstract

Extensive worldwide efforts are underway to produce knockout mice for each of the ~ 25,000 mouse genes, which may give new insights into the underlying pathophysiology of neurological disease. Microscopic magnetic resonance imaging (μMRI) is a key method for non-invasive morphological phenotyping, capable of producing high-resolution 3D images of *ex-vivo* brains, after fixation with an MR contrast agent. These agents have been suggested to act as active-stains, enhancing structures not normally visible on MRI. In this study, we investigated the structural correlates of the MRI agent Gd-DTPA, together with the optimal preparation and scan parameters for contrast-enhanced gradient-echo imaging of the mouse brain. We observed that *in-situ* preparation was preferential to *ex-situ* due to the degree of extraction damage. *In-situ* brains scanned with optimised parameters, enabled images with a high signal-to-noise-ratio (SNR ~ 30) and comprehensive anatomical delineation. Direct correlation of the MR brain structures to histology, detailed fine histoarchitecture in the cortex, cerebellum, olfactory bulb and hippocampus. Neurofilament staining demonstrated that regions of negative MR contrast strongly correlated to myelinated white-matter structures, whilst structures of more positive MR contrast corresponded to areas with high grey matter content. We were able to identify many sub-regions, particularly within the hippocampus, such as the unmyelinated mossy fibres (stratum lucidum) and their region of synapse in the stratum pyramidale, together with the granular layer of the dentate gyrus, an area of densely packed cell bodies, which was clearly visible as a region of hyperintensity. This suggests that cellular structure influences the site-specific distribution of the MR contrast agent, resulting in local variations in T_2_*, which leads to enhanced tissue discrimination. Our findings provide insights not only into the cellular distribution and mechanism of MR active-staining, but also allow for three dimensional analysis, which enables interpretation of magnetic resonance microscopy brain data and highlights cellular structure for investigation of disease processes in development and disease.

## Research highlights

► Investigated preparation and MR parameters for contrast-enhanced mouse brain imaging. ► Also investigated correlates of active-staining for optimised imaging of mouse brain. ► *In-situ* brains scanned with optimised parameters enabled detailed, high SNR images. ► Correlated MR brain structures to histology, detailing fine histoarchitecture visible. ► Our findings may provide insights into the cellular distribution of MR active-stains.

## Introduction

An ever-increasing number of mice are being created through genetic manipulation to advance the goal of translational neuroscience to produce and identify mouse models of human conditions. In order to investigate and validate these mouse models, detailed studies must be undertaken of the mouse brain. Phenotyping the brain conventionally relies on examination of cellular and anatomical structures by histology. While this method is ideal to visualise physiological or pathological changes on a cellular and architectural level, tissue processing artefacts and shrinkage affect the reliability of 3D volumetric assessments. Effective methods for rapid, non-invasive phenotyping are thus needed for the analysis of mouse mutants.

In recent years these efforts have been advanced by the increasing use of microscopic MRI (μMRI) to characterise brain phenotypes of novel mouse mutants. MRI offers a number of advantages over traditional histological sectioning, namely its ability to produce non-invasive datasets, which allow the accurate calculation of volumes without distortion. Morphometric techniques may then be used to allow the identification of novel phenotypes. Work in this field has lead to the creation of non-invasive mouse brain atlases (Dorr et al., 2008; Ma et al., 2005) and has generated new insights into models such as Huntingdon's disease and age-related neurodegeneration (Lerch et al., 2008; Sawiak et al., 2009b; Wetzel et al., 2008).

μMRI produces high resolution datasets of *ex-vivo* tissue with isotropic resolutions of less than 100 μm (typically between 21.5–70 μm in most brain studies (Badea et al., 2007b; Sawiak et al., 2009a). This can be technically challenging, with each voxel representing a tissue volume of < 1 nL, conventionally requiring long scan times to obtain sufficient signal-to-noise (SNR). The innovation of tissue active staining, where gadolinium-chelate MR contrast agents are used during the fixation process to shorten T_1_ relaxation, has enabled the acquisition of high SNR 3D datasets with greatly reduced scan times (Johnson et al., 2002a).

The appearance of μMR images acquired in this manner is sensitive to preparation and scan parameters including the contrast agent, fixation concentration and pulse-sequence used (Cleary et al., 2009). It has been recently shown that the use of higher contrast agent concentrations (> 5 mM Gd-DTPA) as part of the fixation of mouse brains excised from the skull (*ex-situ*), in combination with a 3D gradient-echo sequence, can produce images with high contrast, delineating a number of brain structures seen on conventional histology (Huang et al., 2009; Kim et al., 2009).

The combination of a high contrast agent concentration with *in-situ* preparation may produce enhanced neuroanatomical contrast and allow more detailed and sensitive morphometric atlases, with clearer delineation of regions of phenotypic change. Resulting volume measurements and morphometric analysis may also be less affected by preparation than *ex-situ* brains (Badea et al., 2007a). However, there is little data in the literature comparing *ex-* and *in-situ* brains by MRI. Furthermore, the timecourse for optimal fixation of *in-situ* brains with an MR contrast agent is unknown and a direct histological correlation of brain structures enhanced through gradient-echo imaging together with active-staining has not been performed.

The specific aims of our study were to investigate enhanced tissue contrast using an active-stain μMRI preparation and optimised scanning protocol (Cleary et al., 2009) for imaging adult mouse brains. Specifically we have assessed the effect of active-staining on perfuse- and simple immersion-fixed *ex-situ* brains and monitored the *in-situ* MR tissue characteristics over a period of 9 weeks. Furthermore, we sought to identify the optimal imaging parameters to enable rapid imaging of *in-situ* brains in 3 h with a standard imaging coil. In particular we have assessed contrast-enhanced brain structures via a direct comparison with tissue histology, which may give insights into the cellular distribution and mechanism of MR active-staining.

## Materials and methods

### Animal preparation

Wild-type Tc1 mice (n = 8) (O'Doherty et al., 2005) were taken from a colony maintained by mating Tc1 females to F1 (129 S8 × C57BL/6) males. The mice were culled by an overdose of sodium pentobarbitone administered by injection.

#### *Ex-situ* brain study

In order to investigate the preparation of excised brains we compared the effects of perfusion-fixation to simple immersion-fixation. Two of 4 mice were given an initial saline flush (15–20 ml of normal saline at a flow rate of 3 ml/min introduced via a needle in the left ventricle) and then perfusion-fixed with 50 ml of 4% buffered formol-saline (Pioneer Research Chemicals, Colchester, UK) with 8 mM Gd-DTPA (Magnevist, Bayer-Schering Pharma, Newbury, UK) at a flow rate of 3 ml/min; the remaining 2 mice were simply culled. All 4 brains were then carefully extracted from the skull and post-fixed in 4% buffered formol-saline (with 8 mM Gd-DTPA) at 4 °C.

#### *In-situ* study

For *in-situ* brain MRI, 4 mice were perfusion-fixed as previously described with 2 fixed using formol-saline perfusate alone and 2 with formal-saline with 8 mM Gd-DTPA. After this, mice were decapitated and skin, muscle, lower jaw, tongue, nasal bones and zygomatic arches removed, with the remaining intact skulls post-fixed in 4% buffered formol-saline (with 8 mM Gd-DTPA) at 4 °C.

### Imaging

All imaging was performed on a Varian 9.4 T DirectDrive VNMRS system (Varian Inc., Palo Alto CA, USA) with a 26 mm quadrature birdcage coil (RAPID Biomedical GmbH, Würzburg, Germany). Samples were removed from fixative and excess solution carefully blotted with a paper towel. They were then immersed in Fomblin perfluoropolyether (type PFS-1, Solvay Solexis S.p.A., Bollate, Italy) in 20 ml plastic syringes and immobilised with surgical gauze. Samples were allowed to equilibrate for at least 2 h at room temperature prior to imaging. Air bubbles in samples were minimised by the equilibration time and gentle agitation of the syringe.

#### *Ex-situ* study

T_1_, T_2_* and 3D gradient-echo images of the 4 *ex-situ* brains were obtained after a 7 day fixation. One immersion fixed and 1 perfusion-fixed brain were then reimaged after 2 week fixation and further T_1_, T_2_* and 3D gradient-echo images obtained. Parameters: single sagittal slice 0.5 mm thick), matrix = 128 × 64, FOV = 18 × 9mm, NSA = 4, TR was at least 5 × T_1_. T_1_: inversion-recovery spin echo, 17 TIs (range 6–400 ms), TE = 10 ms; T_2_*: gradient echo, FA = 90°, 11 TEs (2.9–14 ms). 3D RF-spoiled gradient-echo imaging: FOV = 18 × 18 × 9mm, TR = 20 ms, FA = 60°, NSA = 6, matrix = 256 × 256 × 128, TE = 9 or matrix = 450 × 450 × 225, TE = 6 ms.

#### *In-situ* study

Four wild-type Tc1 skulls were fixed and imaged over a 5 week period. T_1_ and T_2_* maps were obtained at each time point. Parameters: single sagittal slice (0.5 mm thick), matrix = 128 × 85 (reconstructed on the console to 128 × 128), FOV = 19.5 × 13mm, NSA = 4. TR was at least 5 × T_1_. T_1_: inversion-recovery spin echo, 13 TIs (range appropriate for estimated T_1_), TE = 10 ms; T_2_*: gradient echo, FA = 90°, 8 TEs (range appropriate for estimated T_2_*). 40 μm isotropic, structural 3D gradient-echo images at unoptimised parameters were also obtained (FOV = 20.48 × 13.04 × 13.04 mm, matrix size = 512 × 326 × 326, TE/TR/FA = 6 ms/20 ms/60°, NSA = 2 or 6). Measured tissue parameter values were input into a Matlab program (The Mathworks Inc., Natick MA, USA) employing the theoretical equation for spoiled gradient echo signal Eq. (1) to determine the approximate optimal scan parameters for maximal cortex–corpus callosum contrast in a 3 h scan-time, as described previously (Cleary et al., 2009).

(1) S‵NR′∝M0sina⋅1−e−TRT11−cosa⋅e−TRT1⋅e−TET2*⋅NSA.

The effect of echo time on brain contrast was also investigated experimentally by repeated imaging of one brain at 5 weeks fixation with 5 different echo-times (3.8–7 ms).

### Delineation of anatomy on MRI and histological correlation

Four additional perfusion-fixed *in-situ* brains were then imaged using our final protocol after 9 weeks fixation (males, ages 18–21 weeks) in 4% buffered formol-saline (with 8 mM Gd-DTPA), using an RF-spoiled gradient-echo sequence. Optimised 3D GE scan parameters: FOV = 20.48 × 13.04 × 13.04 mm, matrix size = 512 × 326 × 326, TE/TR/FA/NSA = 4 ms/17 ms/52°/6.

One brain was carefully extracted from the skull, dehydrated using graded alcohols and xylene, embedded in paraffin, cut into 3-μm sagittal sections and processed for haematoxylin–eosin (H&E) and Luxol Nissl staining. Antibodies or antisera against the following antigens were used: GFAP (DAKO Z0334), MAP-2 (Chemicon MAB3418), Neurofilament 200 (Sigma N5389); and myelin basic protein (SMI94). All immunostaining was carried out using the automated Ventana Benchmark or Discovery (Ventana Medical Systems) automated staining apparatus following the manufacturer's guidelines, using biotinylated secondary antibodies and a horseradish peroxidase-conjugated streptavidin complex and diaminobenzidine as a chromogen. Slides were photographed with a SIS Megaview 3.2 megapixel digital camera mounted on a ZEISS Axioskop.

### Image processing

T_1_ and T_2_* maps were created by a pixel-by-pixel, least-squares fit of the resulting data using in-house C-programs. The inverse of these were also calculated, using ImageJ (National Institutes of Health, Bethesda MD, USA), producing corresponding maps of R_1_ and R_2_*. Statistical analysis of map values was performed in Prism v5.00 (GraphPad Software, San Diego CA, USA). 3D volume images were reconstructed and converted to Analyze format using custom Matlab code and reviewed in ImageJ. SNR and CNR were measured in a number of brain regions by taking the ratio of average and signal differences in whole-structure volumes and the standard deviation in a region of background noise Eq. (2):

(2) $$\begin{array}{cc} SNR=\frac{Signal}{Noise\;SD} & CNR=\frac{Signal_{1}-Signal_{2}}{Noise\;SD} \end{array}\text{.}$$

Visualisation of brain structures was performed in Amira software (v5.2.2, Visage Imaging Inc., Andover MA, USA).

## Results

### *Ex-situ* study: comparison of immersion and perfusion-fixed brains at two fixation durations.

**Fig. 1:**
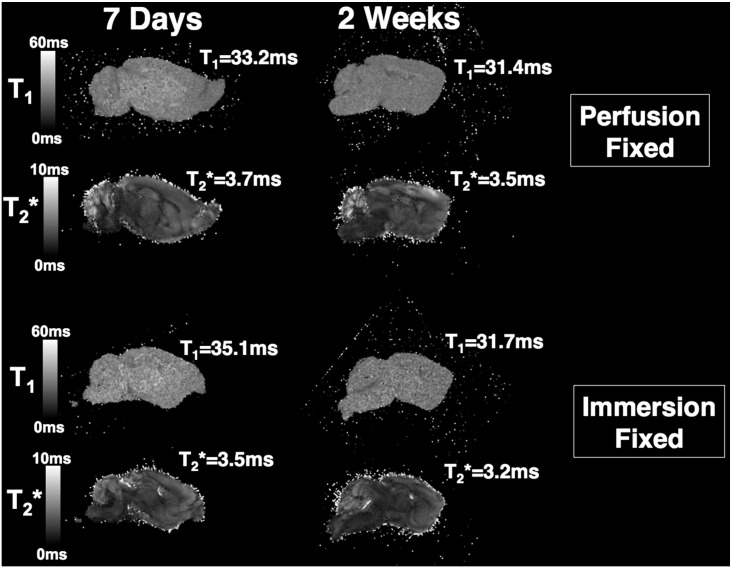
T_1_ and T_2_* maps showing similar sagittal slices and mean, whole-brain T_1_ and T_2_* values through representative immersion and perfusion-fixed brains after 7 days and 2 weeks.

**Fig. 2:**
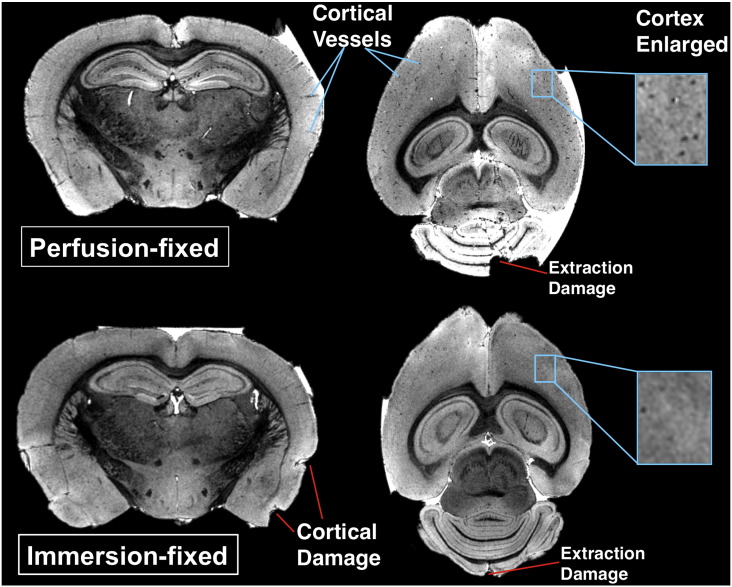
Representative slices through 3D volumes of brains imaged after 2 weeks fixation (40 μm isotropic resolution).

After 7 days immersion in contrast-fixative, there was no significant difference in T_1_ and T_2_* values between the immersion and perfusion-fixed brains (p > 0.40 in all comparisons) (Table 1). T_1_ and T_2_* measurements were repeated at 2 weeks (Fig. 1). We observed a similar change in whole-brain T_1_ (perfusion: 33.2 to 31.4 and immersion: 35.1 to 31.7) and T_2_* (perfusion: 3.7 to 3.5 and immersion: 3.5 to 3.2) between 7 days and 2 weeks (Fig. 1). We noted that the immersion-fixed brains had sustained some additional cortical damage during the extraction process when compared to the perfusion-fixed brain (Fig. 2). Furthermore, cortical vessels, although present in both immersion and fixed brains, were more apparent in the perfusion-fixed brains, otherwise the brains from the two methods appeared similar. This comparison indicates that the use of a simpler immersion-fixation method, where the brain is removed directly from the skull without perfusion-fixation, would facilitate magnetic resonance microscopy techniques, if damage from extraction is not a confounder.

### *In-situ* study

**Fig. 3:**
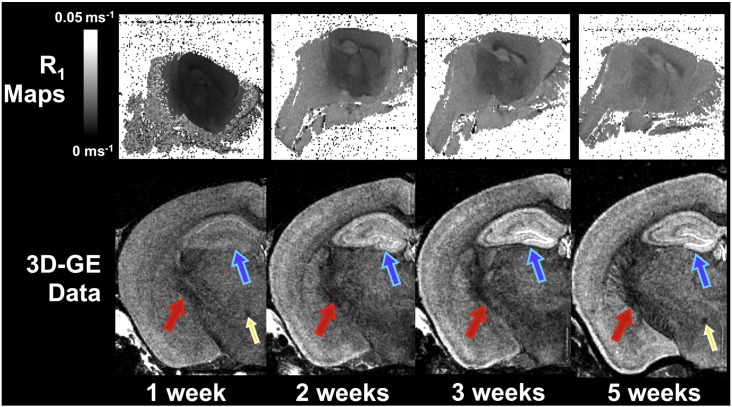
Comparison of representative R_1_ maps and similar volume slices from the same *in-situ* brain over 5 weeks fixation. As seen in R_1_ maps (top) a greater amount of time is required for values in the centre of the brain to equilibrate with more cortical regions. Although a modest SNR enhancement may be seen in 3D gradient-echo images after successive weeks (bottom), there is a noticeable improvement in the quality of structural delineation, particularly in the hippocampus (blue-arrows) and white matter structures (e.g. internal capsule, red arrows; mammilothalamic tract, yellow arrows). (Images identically scaled).

Given that varying degrees of cerebellar and cortical damage was apparent in all brains imaged, we explored the feasibility of contrast-enhanced imaging with the brain *in-situ*. Initially we investigated whether the addition of 8 mM Gd-DTPA in the initial perfusate would improve the rate of T_1_ reduction over perfuse fixation and immersion fixation alone. After measurement of T_1_ and T_2_* values in each group after 1 week fixation, it was apparent that there was no significant difference (p > 0.65) in T_1_ data: therefore all brains were pooled in subsequent analysis. We measured MR parameters over 1, 2, 3, and 5 weeks. Table 2 demonstrates that T_2_* values were found to reach a minimum at 3 weeks immersion (mean thalamus–midbrain = 2.9 ms, cortex = 3.8 ms). In contrast, T_1_ values continued to reduce after 3 weeks, approaching uniformity across the brain by the 5 week timepoint (mean thalamus–midbrain region = 48 ± 3 ms, cortex = 43 ± 2 ms, Table 2). Fig. 3 illustrates the corresponding regional R_1_ changes occurring over 5 weeks. We observed that a greater immersion time lead to a lower T_1_ (e.g. whole-brain T_1_: 1 week 109 ± 53 vs. 5 weeks 44 ± 2 ms, p < 0.05, Table 2) and also reduced the magnitude of T_1_ difference between cortical and midbrain–thalamus structures deep in the centre of the brain (e.g. mean difference at 1 week = 15 ms vs. 5 weeks = 5 ms, Table 2). An increase in the conspicuity of white matter structures (such as fibres of the internal capsule) was observed on 3D images with increasing immersion time (Fig. 3).

### Scan parameter optimisation

**Fig. 4:**
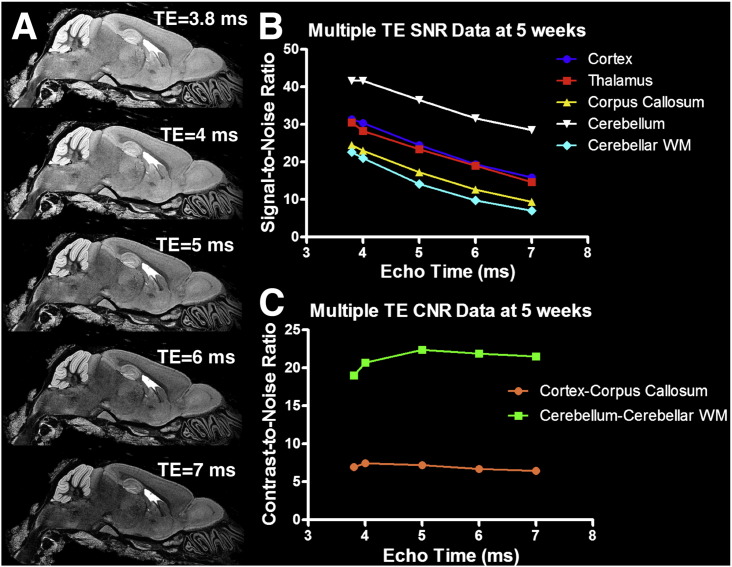
Representative sagittal slice (A) and measured SNR (B) and CNR (C) from 3D volume data of a brain imaged after 5 weeks fixation at five different echo times.

**Fig. 5:**
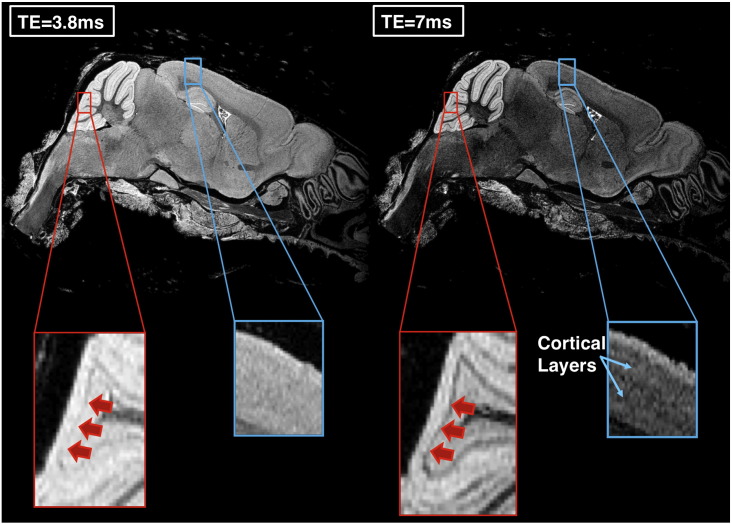
Enlarged sections of cerebellar lobe and cortex showing their appearance at TE = 3.8 and 7 ms. Red arrows appear to correlate to the Purkinje layer of the cerebellum and appear to become more defined at a longer TE, as does the visibility of cortical layers. (Images identically scaled).

From calculations using mean T_1_ across the whole-brain at 5 weeks immersion, we determined that parameters of TR = 17 ms, NSA = 6, at the whole-brain Ernst angle (47°) would produce the highest SNR (assuming constant noise), in a 3 h scan time. From the T_2_* data at 1 week we estimated the ratio of M_0_ in the corpus callosum relative to cortex in the 4 brains to be 0.96 ± 0.01. Using these estimates and T_2_* values from our maps at 5 weeks, we calculated that a TE of approximately ~ 3.4 ms would give optimal cortex/corpus callosum contrast. To ascertain the optimal TE at 5 weeks experimentally, we performed repeated high-resolution scans of a single brain from our dataset with the calculated TR, NSA and flip angle above. Fig. 4 shows the effect of varying TE from 3.8 ms (the minimum permitted by the system at 100 kHz bandwidth) to 7 ms. Although SNR in all structures was highest at TE = 3.8 ms (whole-brain SNR = 31.5), cortex–corpus callosum CNR appeared to be maximal at TE = 4 ms (CNR = 7.5, whole-brain SNR = 30.3). Interestingly white-grey matter CNR in the cerebellum peaked slightly later at a TE = 5 ms (CNR = 22.4). As indicated in Fig. 5, it was noted that increasing echo time appeared to enhance the delineation of more subtle structures, such as cortical layers and the layer of Purkinje cells in the cerebellum, indicating a T_2_* dependency to this contrast. Opting for a balance of high SNR and high grey-white matter CNR, we chose final parameters of TE = 4 ms, FA = 47° (the Ernst Angle), TR = 17 ms and NSA = 6 averages for future studies.

### Brain MR parameters at 9 weeks

**Fig. 6:**
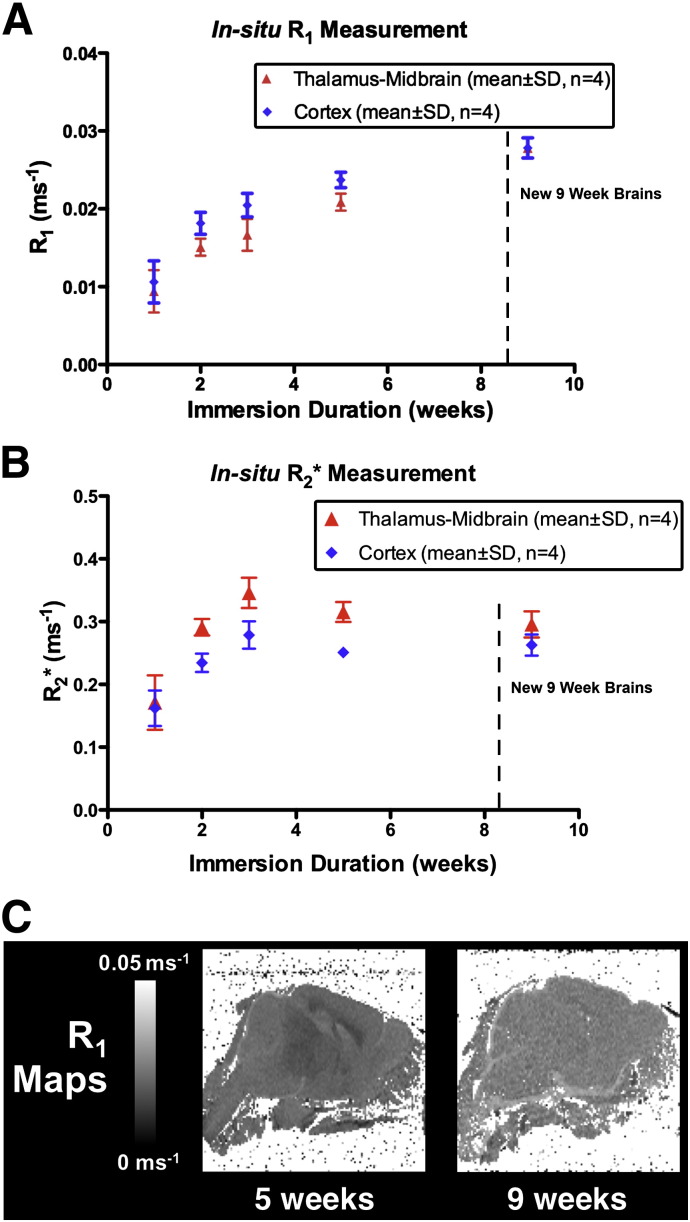
Graph showing the timecourse of R_1_ (A) and R_2_* change (B) with immersion time in fixative + Gd-DTPA solution, incorporating values from the previous 5 week study and 9 week brains indicating a minima has been reached. Representative R_1_ maps from 5 and 9 week brains shown for illustration (C).

After identifying the need for a long immersion duration to enable full penetration of contrast agent, we applied the above optimised parameters (FA = 52°, appropriate for these T_1_s) to a further 4 *in-situ* brains after 9 weeks immersion, acquiring T_1_ and T_2_* maps and high-resolution 3D volumes. Compared to 5 week data, while R_2_* values remained similar in both cortical and thalamus–midbrain regions to our previous brains (Fig. 6B), R_1_ showed a more marked increase (Fig. 6A), with the corresponding whole-brain T_1_ measured as 35 ± 2 ms compared to 44 ± 2 ms of previous brains at 5 weeks. As seen in a corresponding R_1_ map of a sagittal slice through an example brain (bottom right, Fig. 6), values have a narrow distribution across the brain (SD of T_1_ values over whole-brain ROI = 3.8 ± 0.1 ms, n = 4) indicating that 9 weeks immersion may be more suitable. Whole-brain SNR was comparable to previous 5 week data and measured to be ~ 30.

### Structural correlates of active-staining in the brain

**Fig. 7:**
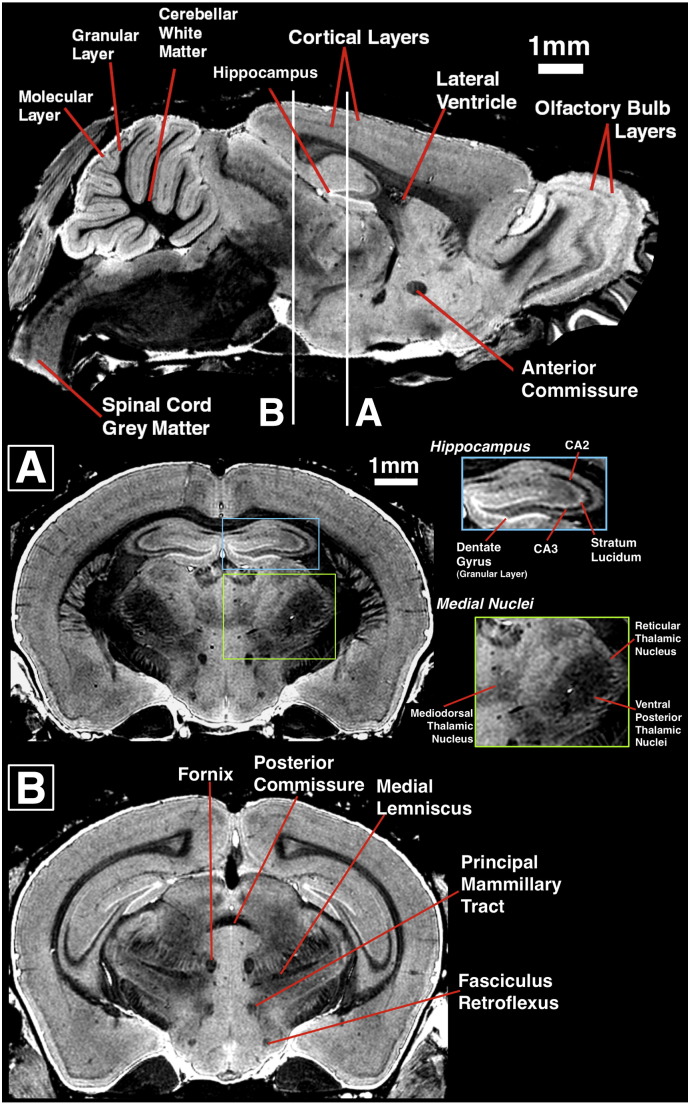
Sagittal and corresponding coronal views through a brain fixed for 9 weeks and imaged at optimised parameters. Sagittal view (top) shows the principal anatomical structures seen. Coronal view (A) shows hippocampal anatomy visible (blue panel) and visible nuclei (green panel). Coronal view (B) shows visible white-matter tracts.

The resulting gradient echo images acquired at the selected parameters (TE/TR/FA/NSA = 4 ms/17 ms/52° Ernst angle/6 averages), after 9 weeks in contrast-fixative, demonstrated excellent contrast, enabling the delineation of a number of structures in the cortex, hippocampus, olfactory bulb and cerebellum (Fig. 7, sagittal image). In the hippocampus, individual layers were clearly visible such as a bright granular cell layer of the dentate gyrus, as well as bright region of the stratum lucidum and dark layer in the region of CA3 (Fig. 7, coronal view A, blue panel). Individual nuclei were also visible, including medial and lateral habenular nuclei and a number of thalamic nuclei (Fig. 7, coronal view A, green panel). White-matter tracts were easily visualised, appearing with strong negative contrast on gradient-echo images (Fig. 7, coronal view B).

**Fig. 8:**
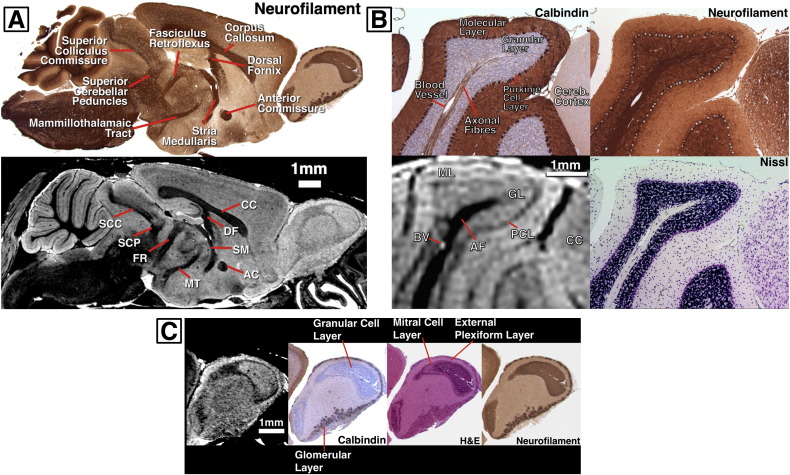
(A) Representative sagittal sections through MRI and a similar neurofilament histology section in the same brain, showing visible white-matter anatomy. (B) Detail through the cerebellum showing the correspondence of structures between histology and MRI such as the granular, molecular and Purkinje layers and axonal fibre tracts. (C) Detail through the olfactory bulb showing layers identified on similar histology sections from the same brain.

Sagittal MR images were compared to histology where a good correlation was observed between a number of white matter structures defined on a neurofilament stained section and equivalent dark areas on a similar MR image (Fig. 8A). Structures such as the anterior commissure, superior colliculus commissure and fasciculus retroflexus were easily visualised.

In the cerebellum, (Fig. 8B) structures on MR correlated well to those seen on histology, such as the bright outer molecular layer, consisting of mostly Purkinje cell dendrites (seen on calbindin section, Fig. 8B), a bright granular cell layer and dark Purkinje cell layer (evenly-spaced, large Purkinje cells visible in all sections, Fig. 8B) and axonal fibre tracts (Fig. 8B, neurofilament section). While MR signal intensity was high in both molecular and granular layers, the granular layer appeared to be slightly hypointense.

In the olfactory bulb (Fig. 8C) the main features of mitral, glomerular and external plexiform layers were easily identified in MR images, with these regions correlating well to all stains. Sublayers in the granular cell layer, seen on H&E and calbindin sections (Fig. 8C), were not readily apparent on MR with images best correlating to the more uniform appearance of the granular cell layer observed in the neurofilament section.

**Fig. 9:**
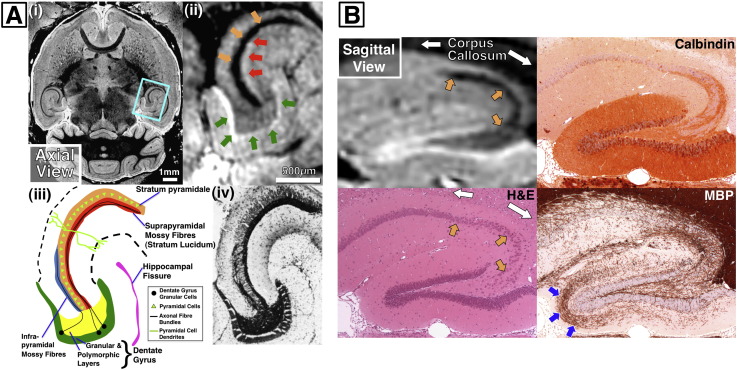
(A) Hippocampal view (ii) from an axial MRI slice (i) compared with a representative section from a C57BL/6 mouse hippocampus stained with Timm silver sulphide stain (iv), showing regions of high ionic zinc concentration, a feature of hippocampal mossy fibres. Red arrows indicate correspondence with mossy-fibre bundles, orange with pyramidal cells and green with the granular cell layer of the dentate gyrus (Timm section adapted from (Crusio et al., 1986). (B) Sagittal view of hippocampal anatomy from a brain fixed for 9 weeks compared with H&E, calbindin and myelin basic protein (MBP) stained sections. Dark regions on MR (orange arrows) appear to correlate with heterogeneous regions containing mossy fibres and pyramidal cells. Corpus callosum (white arrows) indicated for comparison. Blue arrows in the MBP section indicate a diffuse region of myelinated fibres in the molecular layer of the dentate gyrus.

As hippocampal anatomy was especially well defined on MRI, we sought to identify the source of the regional MR contrast by histological comparison. In addition to standard histological stains, we also compared our data to a standard Timm stained section—an established method of visualising the presence of intracellular Zn^2+^ that characterises hippocampal mossy fibres (Haug et al., 1971). Fig. 9A shows a comparison of hippocampal structures with a similar axial Timm section from a C57BL/6 mouse (Crusio et al., 1986). We observed a conspicuous area of hypointensity in the stratum pyramidale region of CA3 (orange arrows, Figs. 9A and B), which correlated with unmyelinated mossy fibres synapsing with pyramidal cells (Figs. 9A(iv) and B, Timm and calbindin sections respectively). Previously, we had observed that hypointensity was associated with myelinated white matter structures (Fig. 8A), which is not the case in the stratum pyramidale. An area of myelinated fibres in the dentate gyrus molecular layer (Fig. 9B, blue arrows) did not result in a hypointensity on MRI. Closely associated and lateral to the stratum pyramidale (concave aspect; red arrows) are the suprapyramidal mossy fibres (running in the stratum lucidum) a region of unmyelinated axonal fibres (Figs. 9A and B, Timm and calbindin sections) that appeared as an area of hyperintensity within CA3. The granular layer of the dentate gyrus (green arrows, Fig. 9A) was clearly visible as a region of hyperintensity that correlated to a dense area of closely packed cell bodies (Fig. 9B, H&E and calbindin sections).

## Discussion

After an initial investigation of *ex-situ* brains which showed a high degree of extraction damage. We have demonstrated that *in-situ* brains prepared in a solution of 8 mM Gd-DTPA and imaged with gradient-echo sequence produced high-resolution (40 μm isotropic), high SNR images, with a 3-hour scan time. We have also shown that structures delineated on MR directly correlate to those defined on histology, such as major white matter tracts and layers of the olfactory bulb and hippocampus. This comparison has then enabled us to assess the effects of tissue microstructure on MR contrast of actively-stained tissue.

### *Ex-situ* brain imaging

*Ex-situ* brain imaging with a gradient-echo sequence has been reported previously (Huang et al., 2009; Kim et al., 2009) and our *ex-situ* brains show T_1_ and T_2_* values are in line with those studies (T_1_ ≈ 30 ms, T_2_ ≈ 7 ms, 10 mM Gd-DTPA, after 4 days immersion in contrast-fixative (Kim et al., 2009)) displaying similar image contrast. Additionally we have shown that these values remain relatively unchanged even after extending the immersion time to two weeks (e.g. mean T_1_ perfusion-fixed went from 33 to 31 ms) to leave more time for Gd-DTPA penetration. We have demonstrated that brains need not be perfusion-fixed and that simple immersion-fixation produced equivalent image quality, thus enabling magnetic resonance histology in laboratories where perfusion-fixation is not readily available. Imaging of extracted, *ex-situ* brain tissue does have some advantages, in that smaller RF coils can be used, improving SNR and a smaller field of view may improve imaging-time (Kim et al., 2009). However the tissue damage observed in our *ex-situ* brains may compromise their use for automated and quantitative morphometric analyses where accurate anatomical volumes and morphology are essential for sensitivity.

### *In-situ* brain optimisation

Given the effect of damage in *ex-situ* imaging we have focussed on *in-situ* brain preparation and MR methods optimisation to produce detailed images of the mouse brain. We demonstrated that full penetration of Gd-DTPA into brain tissue, as assessed by R_1_ values in the cortex and basal ganglia, had equilibrated across the brain by 9 weeks. Indicating that a much greater immersion time is required than in the *ex-situ* brain in order to produce the maximal increase in SNR, and thus image quality, across the brain.

Currently there is no standard protocol for *in-situ* brain preparation and scan parameters, with a number of approaches in current use. A T_2_-weighted 3D fast spin-echo sequence (FSE) with brains fixed in 2 mM gadoteridol (a non-ionic macrocyclic Gd-chelate) for at least 7 days (Tyszka et al., 2006) is able to produce T_2_-weighted images of multiple *in-situ* brains with a 32 μm resolution in a 11.3 h scan, and has been successfully used for morphometric phenotyping (Ellegood et al., 2010) and atlasing (Dorr et al., 2008) studies. While this can offer good T_2_-weighted images with an improved imaging time, the use of an FSE sequence can lead to image blurring and a loss of structural definition (Mulkern et al., 1990).

Partial-Fourier spin-echo has also been successfully used for *in-situ* phenotyping studies giving T_1_-weighted images with 21.5 μm resolution in a ~ 2 h scan time (Badea et al., 2007b). Sharief and Johnson (2006) developed an innovative multi-echo T_2_ sequence where reconstructed images combine data from a number of echo times. This has the effect of improving contrast, especially in T_2_-dependent structures such as in the cortex and brain nuclei. In both these imaging protocols, brains are initially perfusion-fixed with a high concentration (50 mM—assuming a standard 0.5 M clinical solution) of gadoteridol and fixed overnight 10% formalin prior to imaging.

Our results indicate that, in the *in-situ* brain, such short immersion periods may not be sufficient to give maximal T_1_ reduction (and thus SNR gain) over the whole brain. If we assume that a T_1_ of ~ 31 ms (2-week *ex-situ* data) is a likely minima for values after Gd immersion, we nearly achieve this level after 9 weeks (whole-brain T_1_ ~ 35 ms). The need for such an increase in immersion time compared to *ex-situ* brains for Gd penetration is most likely due to the presence of intact meningeal layers and limited entry points for fixative into the skull (e.g. through the foramen magnum and orbits) resulting in a greatly reduced exposed brain tissue surface-area.

Although a virtue of gradient-echo imaging is a short TR and thus reduced acquisition time, this makes it sensitive to T_1_ differences. The heterogeneity seen in R_1_ maps, corresponding to higher T_1_ values in more central structures compared to cortical and cerebellar regions, will thus limit T_1_ recovery, causing signal reduction in these portions of the brain. As the majority of these regions, such as basal ganglia and midbrain contain white-matter fibres, there is likely to be an intrinsically lower signal due to the lower T_2_ of myelinated structures (Counsell et al., 2003). Thus it is important to ensure there is adequate agent penetration to ensure full T_1_ recovery in these regions. Although TR could be increased to compensate, we have previously shown that increased signal averaging is generally preferable to small TR increases to boost SNR (Cleary et al., 2009) and is thus not ideal when imaging with a fixed scan time. The benefits of allowing a greater degree of T_1_ reduction are readily seen in our gradient-echo images taken over 5 weeks (Fig. 5), in which structures of the hippocampus and white matter tracts are more conspicuous by the last 5 week timepoint.

Although active-staining primarily acts to reduce tissue T_1_ due to administration of contrast agent (Johnson et al., 2002b; Petiet et al., 2007) there is also a corresponding reduction in T_2_ (Cleary et al., 2010; Petiet et al., 2007). This may also be exploited to produce image contrast using much shorter TEs than in normal tissue.

In our method, the use of a relatively high contrast agent concentration, leads to a large mean reduction and compression of the whole range of normal tissue T_2_ values. For example, as T_2_* is now ~ 3.8 ms as measured in the cortex (*in-vivo* T_2_ ≈ 45 ms at 9.4 T (Uematsu et al., 2007)), our chosen echo-time of 4 ms results in T_2_*-weighting in that region, allowing cortical layers to be visualised. Similarly, increased T_2_*-weighting, improves visualisation of the Purkinje layer of the cerebellum. Thus an 8 mM Gd-DTPA concentration with a gradient-echo sequence with a relatively short TE and TR combines the potential of a short scan time and a high number of signal averages and an optimal T_2_* weighting to reveal tissue contrast.

The contrast observed in our gradient echo images may be explained by a combination of intrinsic tissue MR relaxation and effect of contrast agent concentration. For example, myelinated white matter is known to have a lower T_2_ than grey matter, due to higher bound water content (Counsell et al., 2003), giving a characteristically darker signal on MR images *in-vivo*, which we also observed in our data. This is probably best explained by contrast agent reducing T_2_ values but still preserving T_2_ differences between tissues, which can be exploited by the correct MR parameters. Yet it has also been suggested that distribution of the agent itself may be enhancing contrast, particularly in non-myelinated structures (Huang et al., 2009). Although this is unlikely to account for our observed white matter contrast: using R_1_ maps as an indication of contrast agent concentration, the lack of regional contrast in maps at 9 weeks does not suggest a selective regional distribution of contrast agent, where higher concentration of contrast agent would be expected to result in area of increased R_1_.

Interestingly we also observed negative contrast in classically unmyelinated regions of the hippocampus that did not appear to depend on the presence of even small traces of myelin (as seen in our MBP-stained section, Fig. 9B, blue arrows). Previous work in the *ex-situ* mouse brain has suggested that ~ 23% of brain volume is inaccessible to the Gd-DTPA indicating that the agent is not distributed uniformly at a cellular level (Huang et al., 2009). Our study reinforces this view, as the dark appearance of these unmyelinated regions are likely to be due to increased local Gd-DTPA distribution and MR susceptibility at a local level. Thus the choice of a gradient echo sequence is ideally suited to detection of such regions, especially those with subtle T_2_* differences.

Both intrinsic contrast and susceptibility could explain the subtly different appearance of molecular and granular layers of the cerebellum on MR. Although the granular layer contains a great number of densely packed cells, as seen on the neurofilament stain, there are still a large number of axonal fibres passing through this region. This could result in the observed hypointensity either through intrinsic relaxation mechanisms where the presence of such myelinated tracts, with a lower T_2_*, leads to a partial volume effect with a slight loss in signal, or the regional cytoarchitecture, comprising a mixture of cell bodies and axons, could affect local Gd-DTPA distribution, causing a susceptibility effect.

Similarly we observed an enhancement of the layer of Purkinje cells with increasing echo time. This layer also contains a mixture of large Purkinje cells surrounded by basket cell axons. However we have observed a large T_2_* dependence on contrast in this region, indicating that susceptibility effects through structural heterogeneity play a greater role, probably due to distribution of contrast agent.

In the olfactory bulb, we observed contrast that delineated the mitral and glomerular but not granular layers. In previous *in-vivo* high-resolution T_2_-weighted images (Boretius et al., 2009) all of these regions were observed including granular layers. It is conceivable that the more complex cytoarchitecture of both glomerular and mitral layers may explain this observation. Glomeruli are relatively large structures that consist of a number of nerve synapses, surrounded by glial cells (Kosaka et al., 1998); also mitral cells have axonal fibres that contribute to the olfactory tract (Shepherd et al., 2007). In contrast, granule cells possess only dendritic processes and are without axons (Price and Powell, 1970). This more complex structure may lead to greater susceptibility effects in the presence of the contrast agent in these regions.

Comparisons with previous diffusion-weighted (Shepherd et al., 2006) and *in-vivo* work (Boretius et al., 2009) may offer some explanation for the enhanced contrast seen in the hippocampus. The area of the stratum lucidum has been seen as hyperintense on diffusion images in the rat (Shepherd et al., 2006) indicating a region of restricted diffusion that may affect the access of Gd-DTPA to the region and thus maintain a longer T_2_* than surrounding tissue. Similarly, the dense packing of granular cells in the dentate gyrus may prevent infiltration of contrast into the region and also maintain a long T_2_* resulting in its bright appearance.

Previous work (Benveniste et al., 2000) has shown contrast enhancement in the hippocampus on subsequent *ex-vivo* images monitored over a period of 2 to 50 h after initial *in-vivo* perfusion-fixation with Gd-DTPA. Images acquired at 2 h post fixation showed a general increase in signal over the whole hippocampus, but the granular layer of the dentate gyrus appeared particularly hyperintense. The stratum pyramidale was visible as a dark band. Over subsequent timepoints, while signal enhancement over the whole hippocampus appeared to be maintained, the conspicuity of both granular layer and stratum pyramidale was greatly reduced. This evolution of contrast was attributed to the vascular nature of this region, with intravascular retention and extravasation of contrast agent leading to the appearance of reduced and increased signal respectively. The initial enhancement seen in the granular layer may indicate that the hippocampus is compartmentalised, possessing regions accessible to the contrast agent to a greater or lesser degree. High-resolution *in-vivo* imaging, without contrast, has also demonstrated delineation of hippocampal structures in T_2_-weighted images, including the stratum pyramidale, and granular and polymorphic layers of the dentate gyrus (Boretius et al., 2009). In images with strong weighting (TE = 82 ms), contrast in these regions was markedly different to that of ours as both dentate gyrus and stratum pyramidale appeared hypointense, with the stratum pyramidale visible as a continuous line from CA3 to CA1. Also the granular layer of the dentate gyrus was not easily distinguished from the polymorphic layer. These findings suggest that in certain structures local accumulation of the contrast agent can contribute to MR appearance more than intrinsic tissue T_2_* contrast. This suggests the exciting possibility that targeting such regions could provide the basis for the investigation of novel MR microscopy stains.

While the presence of contrast agent is likely to contribute most to changes in tissue parameters, an additional consideration is tissue fixation. Aldehyde fixatives cause the cross-linking of proteins (Fraenkel-Conrat and Olcott, 1948), reducing water mobility, and resulting in a reduction of tissue MR relaxation times (Dawe et al., 2009). A study in the rat cortical slice model (Shepherd et al., 2009) found T_1_ reduced 21% after > 10 day fixation, although an 81% T_2_ reduction could be reversed after washing out the fixative by immersion in PBS. Timecourse studies in fixed human brain have also shown progressive decreases in T_1_ and T_2_, with the T_1_ change occurring fastest over the initial 1–2 weeks and a slower decline after 5 weeks (Tovi and Ericsson, 1992). Grey and white matter T_1_ (Pfefferbaum et al., 2004) and T_2_ values (Blamire et al., 1999) also tend to converge with a longer fixation time. There are, however, few studies in the literature assessing the effect of fixation on the use of Gd-chelates. Work in *Xenopus* oocytes (Purea and Webb, 2006) indicates that fixation may alter cell membrane structure, enabling the entry of contrast into the cytoplasm. However, further experiments are needed to examine the role of fixation on contrast in active-stained μMRI of the mouse brain.

The time needed for the penetration of agent in our methodology may be a disadvantage, as histological techniques, especially in light of developments in automatic sectioning systems, can provide more detailed information in a similar amount of time. However, MRI does offer the benefits of non-destructive 3D image acquisition.

## Conclusion

We have shown that active-stained μMRI can provide detailed anatomical images of the *in-situ* mouse brain with high resolution and signal-to-noise. Additionally, we have used a variety of stains to identify and compare the MRI appearance of brain structures to those defined by histology. Combining this data, we have highlighted possible mechanisms for the enhanced contrast in these regions, surmising that contrast depends on both preserved, intrinsic T_2_* differences as well as regions delineated by distribution of active-stain due to tissue microstructure. This methodology could enable greater sensitivity for the phenotypic characterisation in mutant mouse models and enhance regions of the mouse brain that may be targeted in future transgenic studies.

## Figures and Tables

**Table 1:** Table showing T_1_ and T_2_* values in brains after 7 days immersion only or with initial perfusion-fixation (mean values ± SD).

Region of interest	Perfusion Fixation (n = 2)			Immersion fixation (n = 2)		
Cortex	Cerebellum	Whole-brain	Cortex	Cerebellum	Whole-brain
T_1_ (ms)	32.2 ± 0.4	31.8 ± 0	33.2 ± 0.2	33.1 ± 1.3	28.2 ± 6.2	34.2 ± 1.3
T_2_* (ms)	4.4 ± 0.6	5.1 ± 0.8	3.7 ± < 0.1	4.4 ± 0.2	5.2 ± 0.2	3.8 ± 0.3

**Table 2:** Table showing T_1_ and T_2_* values within cortical and central thalamus–midbrain regions of interest over 5 weeks fixation.

(n = 4)	T_1_ (ms)		T_2_* (ms)	
Region of interest	Thalamus–midbrain	Cortex	Thalamus–midbrain	Cortex
*Immersion duration*				
1 week	113 ± 32	99 ± 23	6.1 ± 1.5	6.3 ± 1.1
2 weeks	67 ± 5	55 ± 4	3.4 ± 0.2	4.3 ± 0.3
3 weeks	61 ± 7	49 ± 3	2.9 ± 0.2	3.6 ± 0.3
5 weeks	48 ± 2	42 ± 2	3.2 ± 0.2	4.0 ± 0.1
